# Social and Poor-Law Problems

**Published:** 1906-07-28

**Authors:** 


### SOCIAL AND POOR LAW PROBLEMS.

### REGIMENTAL HOMES FOR INVALID SOLDIERS.

In the year 1901, as a memorial to the late Prince Christian Victor of Schleswig-Holstein, a scheme was planned of building Cottage Homes for invalid soldiers in the neighbourhood of the headquarters of the various regiments. The memorial was closed in 1902, but the work initiated by it has been carried on by the "Regimental Homes and Benefits Agency" under the secretaryship of the same lady, Mrs. Papillon, and there has therefore been no interruption of it. The idea of these Homes—the one which differentiates them from other hospitals for old soldiers—is that they should be within the territorial limits from which the regiment takes its name, that the inmates should be, of course, men who have served in that regiment, and that they should be elected to their tenancy of the Home by the votes of their former comrades. This last regulation is a great satisfaction to the fortunate veterans who get into the Homes, as it takes away from them the notion of charity, and places the election upon the basis of recognition of what they have done and suffered. Each cottage is a real home, where the old soldier can take his wife and family, and it is always under the care of some lady or gentleman of the neighbourhood, who will help him to obtain any work he is fit for. There are now 32 of these regimental Homes, and it is noteworthy that these retreats for those who have suffered in their country's service form a good recruiting ground. Each has in some prominent place the flag or badge of the regiment it belongs to, recruiting notices are posted at the gate, and beyond these palpable advertisements there is the moral effect on brave and ardent youth of the conversation of the veteran who "shoulders his crutch and shows how fields were won." Colonel Gildea, the Chairman of the Central Committee of the Homes, says that there can be no question of the popularity of the scheme with the soldier, while he adds, "the civilian view is that this regimental provision will add to the popularity of the Army; and the civilian knows that the popularity of the Army is all that stands between him and conscription." Some of these regimental Homes have been founded, and more or less completely endowed, by those who have friends in the Army, often as a memorial to those they have lost. In others the regiments themselves have made the first contribution to the establishment of their Home, but officers are not all men of means, and it cannot be expected that this source alone will suffice for the building, furnishing, and maintenance of the cottages. Thus there must be an appeal to the public at large. Those who love and admire our soldiers, as we all did in the days—not so long ago!—of the South African war, should be willing to do something towards the founding of these honourable retreats for old soldiers and their families. And if there be any who will not give for love, let them take to heart Colonel Gildea's dictum, "The popularity of the Army is all that stands between the civilian and conscription," and for their own sakes assist in a truly noble work which has the indirect result of also making the Army popular. Those who have an interest in a special regiment can give their contribution to that regiment's Home; others can give through the Regimental Homes and Benefits Agency, the offices of which are at 11 Tothill Street, Westminster, feeling sure that their gift will help to make more comfortable the days of those who have stood between us and our enemies, and lost health and strength in the task.

